# Periodic orbits in chaotic systems simulated at low precision

**DOI:** 10.1038/s41598-023-37004-4

**Published:** 2023-07-14

**Authors:** Milan Klöwer, Peter V. Coveney, E. Adam Paxton, Tim N. Palmer

**Affiliations:** 1 Atmospheric, Oceanic and Planetary Physics, University of Oxford, Oxford, UK; 2 Earth, Atmospheric and Planetary Sciences, Massachusetts Institute of Technology, Cambridge, MA USA; 3 Centre for Computational Science, University College London, London, UK; 4 Advanced Research Computing Centre, University College London, London, UK; 5 Informatics Institute, University of Amsterdam, Amsterdam, The Netherlands

**Keywords:** Atmospheric science, Information theory and computation, Mathematics and computing, Applied mathematics, Computational science, Computer science

## Abstract

Non-periodic solutions are an essential property of chaotic dynamical systems. Simulations with deterministic finite-precision numbers, however, always yield orbits that are eventually periodic. With 64-bit double-precision floating-point numbers such periodic orbits are typically negligible due to very long periods. The emerging trend to accelerate simulations with low-precision numbers, such as 16-bit half-precision floats, raises questions on the fidelity of such simulations of chaotic systems. Here, we revisit the 1-variable logistic map and the generalised Bernoulli map with various number formats and precisions: floats, posits and logarithmic fixed-point. Simulations are improved with higher precision but stochastic rounding prevents periodic orbits even at low precision. For larger systems the performance gain from low-precision simulations is often reinvested in higher resolution or complexity, increasing the number of variables. In the Lorenz 1996 system, the period lengths of orbits increase exponentially with the number of variables. Moreover, invariant measures are better approximated with an increased number of variables than with increased precision. Extrapolating to large simulations of natural systems, such as million-variable climate models, periodic orbit lengths are far beyond reach of present-day computers. Such orbits are therefore not expected to be problematic compared to high-precision simulations but the deviation of both from the continuum solution remains unclear.

## Introduction

Many natural systems exhibit chaotic dynamics. The chaos in weather prevents reliable forecasts beyond one or two weeks^1,2^. The turbulent flow of air or water around vehicles requires complex numerical simulations to optimise the drag^3,4^. Similarly, chaos is present in models of many-body problems from astrophysics^5,6^, classical molecular dynamics and chemical reaction networks^7^, and plasma in fusion reactors^8,9^. As chaotic dynamics often prevent analytical solutions, numerical simulations with finite-precision floating-point numbers^10^ are used to approximate a system’s state and predict its future.

However, simulating a deterministic, yet chaotic system with deterministic finite-precision numbers always results in closed periodic orbits due to a finite set of possible states^11^. One may think of these orbits as *bitwise* periodic, in the sense that they eventually return to a state in which every bit of every independent variable is identical to the initial state. Eternal periodicity in the simulation of chaotic systems violates a fundamental property of chaos, but periods are very long with the high precision of 64-bit floating-point numbers (Float64).

Unstable periodic orbits are the *skeleton of chaos*^12^ and have been intensively studied to better understand the dynamical properties of chaotic systems^13–15^. Chaotic trajectories follow a given periodic orbit in its vicinity but eventually diverge due to the orbit’s instability and approach another orbit until diverging again^16^. The spectrum of the periodic orbits is a decomposition of the attractor^17^. It contains infinitely many countable orbits: the more orbits the longer the period with no upper bound on the period length^16,18^. The spectrum is truncated with deterministic finite-precision to a finite set of orbits bounded by a maximum period length. How such a truncation degrades the simulated dynamics is generally unclear. While very low-precision arithmetic truncates chaotic attractors to fixed points or short loops, the degradation can also be less obvious: it may be either substantial or to the point of being negligible.

Computed dynamics have errors relative to analytical solutions. Model errors arise from the difference between the mathematical equations and the natural systems they represent, including unresolved processes and heuristic parameters. Errors in the initial or boundary conditions are a result of imperfect observations being assimilated into the numerical model^19^. Discretization errors occur when a continuous system is discretized into a number of variables that is often limited by available computational resources^20^. In addition, there are rounding errors as a result of using finite-precision numbers to approximate real numbers^21^. In a chaotic system, errors grow exponentially with time, so the largest source of error masks smaller ones. Due to often negligible rounding errors, many numerical simulations are currently transitioning to low-precision calculations in exchange for computational performance^22–24^. The performance gain is then reinvested into a higher resolution or complexity with more independent variables, typically with the intention of increasing the model’s accuracy.

16-bit low-precision computations are increasingly supported on modern processors, such as graphics processing units^25^ (GPU), tensor processing units^26^ (TPU) and also conventional central processing units^27,28^ (CPU). While the standard and only widely available number format are floats, several alternatives have been proposed: posits^29^, logarithmic fixed-point numbers^30^ (logfix), and floats with stochastic rounding^31–33^. Currently lacking in hardware support, these number formats are first emulated in software for precision tests. The comparison across formats provides a better understanding as to how the numerical precision affects the simulated dynamics.

Here, we compare the periodic orbits and invariant measures as the properties of three chaotic dynamical systems when simulated with different binary number formats and rounding modes and at various levels of precision. The number formats are briefly described in section "Periodic orbits and number formats" along with our methodology for finding periodic orbits. In section "The logistic map with various number formats" we analyse the bifurcation of the logistic map as simulated with different number formats and rounding modes. In section "Revisiting the generalised Bernoulli map", we revisit the simulation of the generalised Bernoulli map to analyse numerical precision in a system where the analytical invariant measure is known. In section "Orbits in the Lorenz 1996 system", we turn to the Lorenz 1996 system to investigate the periodic orbit spectrum with an increasing number of variables. Section "Conclusions" summarises the results, section "Methods" provides further details on the methodology.

## Periodic orbits and number formats

The state of a deterministic dynamical system is entirely determined by $X = \left( {x_{1} ,x_{2} ,...,x_{N} } \right)$, the vector of all its $N$ prognostic variables in a given finite-precision number format at a given time step $t$. We define a periodic orbit in a simulation when the state vector $X_{{t_{0} }}$ at time step $t_{0}$ exactly reoccurs at a later time step $t_{1} > t_{0}$,

1 $$X_{{t_{0} }} = X_{{t_{1} }} .$$

Equality is hereby required within the considered precision of the number format. Equivalently, $X_{{t_{0} }}$ and $X_{{t_{1} }}$ are bitwise identical. An exception occurs for floats where $- 0 = 0$, which arithmetically does not impact on the dynamical system. This bitwise periodicity is in contrast to other studies investigating quasi-periodic orbits^34,35^, which require $X_{{t_{0} }} ,X_{{t_{1} }}$ to be close, but not bitwise identical. The periodic orbits here are found in long simulations when Eq. (1) holds and are very sensitive to the choice of the number format and numerical precision. This is distinct from unstable periodic orbits, numerically found via an iterative Newton method when Eq. (1) holds up to a numerical error^36^ that is larger than the precision of the number format.

The invariant measure of a chaotic system describes its attractor independent of the initial conditions. A deterministic chaotic system simulated with deterministic finite-precision arithmetic will converge to one of the periodic orbits for any initial condition. Based on all periodic orbits we can compute the invariant measure through a weighted average by the orbits’ respective basins of attraction, i.e. the fraction of initial conditions that end up on a given periodic orbit. To find *all* periodic orbits in a deterministic dynamical system, all possible initial conditions have to be integrated and checked for periodicity. For a 1-variable system with $X \in \left[ {0,1} \right)$ (such as the Bernoulli map, see section "Revisiting the generalised Bernoulli map") simulated with Float32 there are 1,065,353,216 unique initial conditions. For any larger system or higher precision number format it becomes computationally virtually impossible to consider all initial conditions. We therefore use a Monte Carlo-based random sampling of the initial conditions to find a subset of all orbits. An improved pseudo-random number generator for floats is developed for this purpose (see Methods). The orbits found are expected to be those with the largest basins of attraction, and a robust estimate of their size is obtained for a sufficiently large sample of initial conditions. This procedure is explained in the section Methods.

Floating-point numbers are standardised following IEEE^10,37^. Other binary number formats are briefly introduced. Posits^29^ have a slightly higher precision within the powers of 2 around $\pm 1$, yet a wide dynamic range at the cost of a gradually lower precision away from $\pm 1$. While posits have been proposed as a drop-in replacement for floats, they currently lack widely available hardware support. For more details on posits see refs.^29,38–40^. Logarithmic fixed-point numbers have received little attention apart from research implementations on custom hardware^30^ and are therefore described with our design choices in more detail.

### Logarithmic fixed-point numbers

The logarithmic fixed-point number (logfix) format LogFixPoint16 is here defined with a similar range-precision trade-off as Float16 with a sign bit $s$, $n_{i} = 5$ signed integer bits in the exponent and $n_f = 10$ fraction bits. A logfix number $x$ is of the form

2 $$x = \left( { - 1} \right)^{s} \cdot 2^{k}$$

The exponent $k = i + f$ is equivalent to a fixed-point number with $n_{f}$ binary digits accuracy. The signed integer $i$ is in $\left[ { - 2^{{n_{i} }} ,2^{{n_{i} }} - 1} \right]$ and allows a similar range of representable numbers, 2^-16^ to 2^16^, compared to the exponent bits in Float16. The fraction $f$ is in $\left[ {0,1} \right)$ and identically defined to the mantissa bits in floating-point numbers (without the hidden bit), which are encoded as the sum of powers of two with negative exponent. Multiplication, division, square root and power are easily implemented for a logarithmic number format with binary integer arithmetic and do not introduce any rounding errors unless the result is beyond the range of representable numbers. In contrast, addition and subtraction with logfixs is based on the Gaussian logarithms, which often introduce a rounding error where floats avoid them due to their piecewise uniform distribution. For more details and a software implementation see LogFixPoint16s.jl^41^**.**

### Stochastic rounding

The default rounding mode for floats, posits and logfixs is round-to-nearest^10^, which rounds an exact result $x$ to the nearest representable number $x_{i}$. Stochastic rounding has recently emerged as an alternative rounding mode, beneficial for scientific computing^31,33,42,43^. In this rounding mode $x$ is rounded down to a representable number $x_{1}$ or up to $x_{2}$ at probability proportional to the distance between $x$ and $x_{1} ,x_{2}$

3 $$round_{stoch}(x) = \begin{cases} x_1  \,\;\text{with probability}  \;1 - u^{ - 1} (x - x_1)\\ x_2 \,\; \text{with probability} \; u^{ - 1} (x - x_1) \end{cases}.$$

with $u$ being the distance $x_{2} - x_{1}$ and $x_{1} \le x \le x_{2}$. While deterministic rounding introduces a rounding error at most $\pm \frac{u}{2}$, the stochastic rounding error is bounded by $\pm u$. This occurs at low probability when $x$ is very close to a representable number but rounded away from it. However, stochastic rounding is exact in expectation^32,44^, such that for repeated rounding the expectation of the rounded result converges to the exact $x$. Depending on the algorithm, the effective precision of a number format with stochastic rounding can therefore be higher than with deterministic round-to-nearest^33,42^, despite the same number of mantissa bits. For more details and the software implementation used here see the software package StochasticRounding.jl^45^.

## The logistic map with various number formats

The 1-variable logistic map is one of the most well-known chaotic maps, famous for its period-doubling bifurcation^46^. Starting with $x_{i} \in \left[ {0,1} \right)$ at time step $i = 0$ the logistic map with parameter $r$ is

4 $$x_{i + 1} = rx_{i} \left( {1 - x_{i} } \right)$$

**Figure 1:**
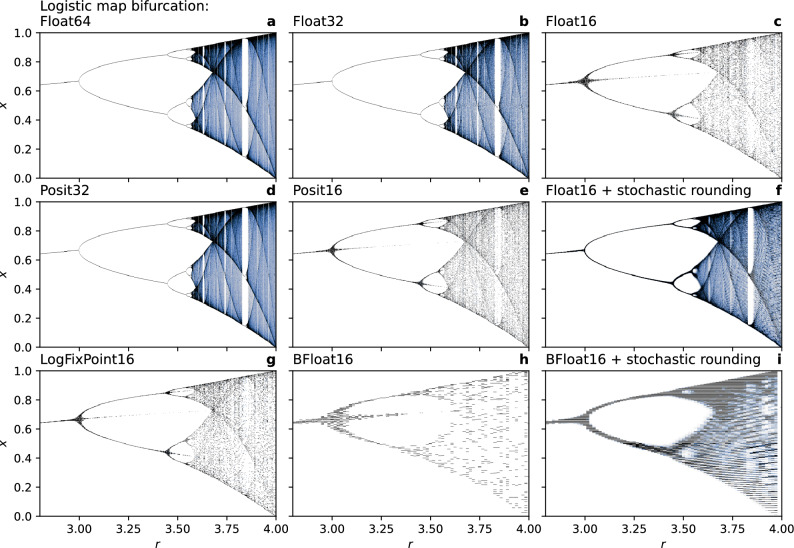
The bifurcation diagram of the logistic map simulated with various number formats, precisions and rounding modes. Bifurcation in the logistic map is a function of its parameter $r$. Shading represents a histogram of the solutions with darker colours denoting higher frequencies. Number formats, precisions and rounding modes in (**a**–**i**) as described in the respective titles.

For $r = \left[ {1,3} \right]$ the system has a stable fixed point at $\frac{r - 1}{r}$ which all solutions will eventually converge to. For $r = 3$ to $r \approx 3.45$ the solution will oscillate between two values, for larger $r$ this period doubles first to 4 time steps, then to 8, 16, … and so on (Fig. 1a). For each period doubling the fixed points before the previous bifurcation continue to exist, but are now unstable. For $r \approx 3.57$ and larger, chaotic solutions are found. However, intermittent ranges of $r$ (often called islands of stability) exist which again have periodic solutions.

These properties of the logistic map are well simulated with Float64, Float32 and Posit32 (Fig. 1a,b,d) due to sufficiently high precision. However, at lower precision with Float16, Posit16, LogFixPoint16 and BFloat16 the unstable fixed points gain stability due to rounding errors (Fig. 1c,e,g,h, shown as third branch at bifurcations). Within the vicinity of these fixed points a diverging solution can be rounded back towards the unstable fixed points, which therefore become spuriously part of the simulated attractor. Furthermore, the chaotic solutions of the logistic map collapse into periodic orbits such that the dense attractor is only approximated by a low number of finite points (shown as sparsely dotted areas in the bifurcation diagram).

Stochastic rounding removes the aforementioned spurious stability of the unstable fixed points due to rounding errors at low precision (Fig. 1f,i). For Float16, stochastic rounding considerably improves the bifurcation diagram over deterministic rounding. Particularly the islands of stability reemerge and the chaotic solutions have a much denser attractor. While the bifurcation diagram with BFloat16 is poorly simulated with both rounding modes, stochastic rounding is still a clear improvement over deterministic rounding.

## Revisiting the generalised Bernoulli map

The generalised Bernoulli map^11^ (also sometimes called the beta-shift^47^ or the Renyi map^48,49^) is a 1-variable chaotic system starting with $x_{i} \in \left[ {0,1} \right]$ at time step $i = 0$ with the parameter ${\upbeta } > 1$ defined as

5 $$x_{i + 1} = f_{\beta } \left( {x_{i} } \right) = \left( {\beta x_{i} } \right){\text{ mod }}1$$

The modulo-operation $\text{mod}$ satisfies that $x \in \left[ {0,1} \right)$ at all future time steps. We note in passing that the generalised Bernoulli map is topologically conjugate to various other dynamical systems, including the aforementioned logistic map. Simulating this system with Float32 was found not to represent the periodic orbit spectrum well^11^, which is in turn closely related to the simulated invariant measure. For the generalised Bernoulli map the analytical invariant measure is known as^50^

6 $$h_{\beta } \left( x \right) = C\mathop \sum \limits_{j = 0}^{\infty } \beta^{ - j} \theta \left( {f_{\beta }^{j} \left( 1 \right) - x} \right)$$

The Heaviside function is $\theta$ and $f_{\beta }^{j} \left( 1 \right)$ is the $j$-th time step of the Bernoulli map starting from $x_{0} = 1$. $C = 1$ is a normalisation constant, but for the calculation of Wasserstein distances (see section Methods) renormalization is applied so that the integral of $h_{\beta } \left( x \right)$ over $\left[ {0,1} \right]$ is equal to 1 and $h_{\beta } \left( x \right)$ a probability density. To better visualise the invariant measure for varying $\beta$ we introduce a normalisation $\widetilde{h_\beta} = h_\beta(x) ~/~ \text{max}(h_\beta(x))$, which is always in $\left[ {0,1} \right]$ and can be applied to the analytical invariant measure as well as simulated ones.

We are revisiting the generalised Bernoulli map with various number formats and rounding modes to better understand a previously suggested pathology^11^ as a function of arithmetic precision. While simulating the Bernoulli map numerically with a given number format, we perform both the multiplication and the subtraction in Eq. (5) with that format and avoid any conversion between number formats. This is in contrast to Boghosian et al. 2019, whose implementation converts $x_{i}$ to Float64 before multiplication with $\beta$ (as Float64) and possible subtraction with 1 (as Float64), i.e. $x_{i + 1} = {\text{Float32}}\left( {\beta {\text{ * Float64}}\left( {{\text{x}}_{{\text{i}}} } \right)} \right){ }~\text{mod}~1$. While hardware allows for fused multiply–add operations without intermediate rounding error, similar to the conversion to Float64 here, the fused conditional subtraction in the modulo is generally not supported on hardware.

### The special $\beta = 2$ case

Boghosian et al. 2019 highlight that the Bernoulli map with $\beta = 2$, and similarly for every even integer, will collapse to $x = 0$ after $n$ time steps with any float format at arbitrary high but finite precision, where $n$ is smaller than the number of bits. The subtraction with 1 acts as a bitshift towards more significant bits, pushing zero bits into the mantissa until $x = 1$ and the modulo returns $x = 0$ (Figure S3a, b and c). This phenomenon occurs as the Bernoulli map with $\beta = 2$ (and similarly for larger even integers) does not introduce any arithmetic rounding error: Both the multiplication with $\beta$ and subtraction with 1 are exact with floats (and also with posits). The multiplication with $\beta = 2$ is exact as the base-2 exponent is simply increased by 1. The subtraction with 1 is exact as every finite positive float or posit can be written as $2^{e} \left( {1 + f} \right)$ for some integer $e$ and a sum $f \in \left[ {0,1} \right)$ of powers of two with negative exponents. Constraining the range to $\left[ {1,2} \right)$, where the subtraction is applied, yields $e = 0$ and so subtracting 1 from the mantissa $1 + f$ is $f$, again a sum of powers of two, which is exactly representable with floats or posits.

The only occurring rounding error is in the initial conditions. While a randomly chosen $x \in [0,1)$ at infinite precision will have infinitely many non-zero mantissa bits, at finite precision those beyond the resolved mantissa bits are rounded to 0. Therefore, the least significant mantissa bit remains 0 after each iteration of the Bernoulli map while the same 0 bit from the previous iteration is further bit-shifted in. This behaviour holds for floats and posits, but it does not occur with logfixs. All multiplications are exact with logfixs (unless under or overflows occur), but in contrast to floats and posits a rounding error occurs in the subtraction, with the possibility of setting the least significant mantissa bit to 1. This rounding error is effective at preventing a collapse of the attractor (Figure S3d). The Bernoulli map with $\beta = 2$ and simulated with floats or posits is therefore special, as it is a chaotic system that does not involve any arithmetic rounding errors beyond the rounding of the initial conditions. However, the simulation of most other systems, including the generalised Bernoulli map with $1 < \beta < 2$, involves rounding errors with any finite precision number format.

### Bifurcation of the invariant measure

Given that the analytical invariant measure is known for the generalised Bernoulli map (Eq. 6), we can assess its representation with various number formats at different levels of precision. Boghosian et al. 2019 conclude that the invariant measure with Float32 is an inaccurate approximation of the analytical invariant measure. While this difference is even more pronounced with Float16 arithmetic (Figure S2), with Float64 the invariant measure is comparably accurate. The question therefore arises as to whether the discrepancy of the invariant measures vanishes with higher precision, or whether a pathology persists at any precision level for some $\beta$.

**Figure 2:**
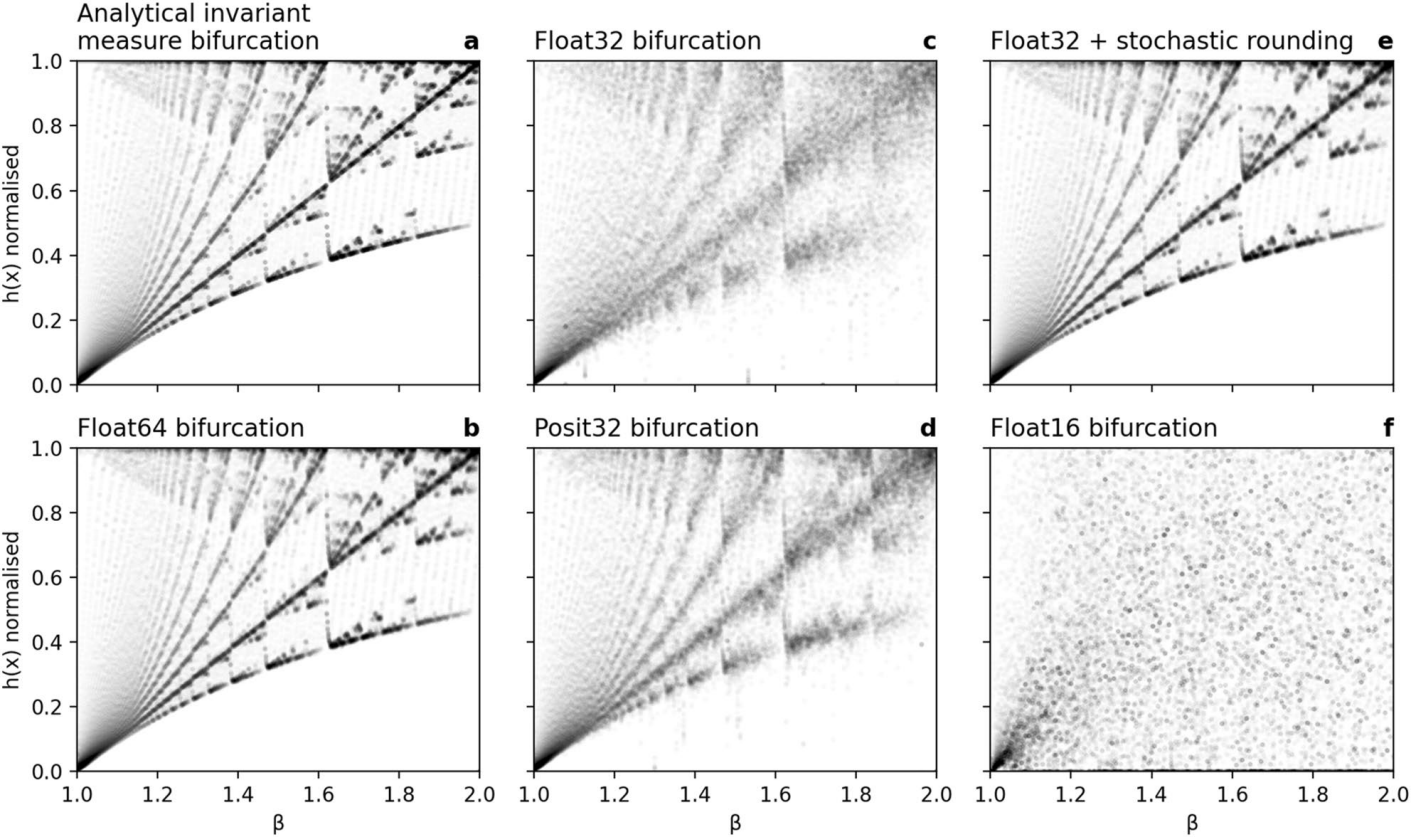
Bifurcation of the quantization levels corresponding to the invariant measure in the generalised Bernoulli map as simulated with various number formats. (**a**) Analytical bifurcation ${h}_{\beta }\left(x\right)$ from the exact invariant measure, normalised by $\text{max}(h_\beta(x))$, compared to the invariant measure by simulating the Bernoulli map with (**b**) Float64, (**c**) Float32, (**d**) Posit32, (**e**) Float32 + stochastic rounding, and (**f**) Float16.

The analytical invariant measure of the generalised Bernoulli map consists of many step functions taking values on a discrete set of points (Figure S2), which bifurcate with increasing $\beta$ (Fig. 2a). These “quantization levels” cannot be exactly represented with Float16 or Float32 arithmetic (Figure S2), such that their bifurcation is visually blurred (Fig. 2c). The visually sharp representation of this bifurcation of the quantization levels with Float64 indicates a much more accurate approximation to the analytical invariant measure (Fig. 2b). Due to the higher precision of 32-bit posit arithmetic (Posit32) around $\pm 1$, the bifurcation is slightly improved with Posit32 over Float32 (Fig. 2d). Given the inaccurate representation of the invariant measure with Float16 (Figure S2), its bifurcation has little resemblance to the analytical bifurcation (Fig. 2f).

### Effects of stochastic rounding

Augmenting Float32 with stochastic rounding considerably improves the bifurcation (Fig. 2e) and makes it virtually indistinguishable from Float64 or the analytic bifurcation. However, stochastic rounding does not decrease the rounding error accumulated over many iterations in a forecast (Figure S4), such that the effective precision is not increased over deterministic rounding. But introducing stochasticity prevents the convergence onto periodic orbits, which are otherwise present with deterministic rounding^11^. Previously inaccessible regions of the attractor can be reached with stochastic rounding as the simulation is frequently pushed off any periodic orbit. This advantage of stochastic rounding is also observed in the logistic map (section "The logistic map with various number formats"). While periodic orbits are not fully removed from solutions due to the use of pseudo random number generators (PRNG) that are themselves periodic, the periods of PRNGs are usually so long that effectively any periodicity is avoided. The period length of Mersenne Twister^51^, the most widely-used PRNG, is $2^{19937} - 1$ and still sufficiently long with $2^{128} - 1$ for the faster Xoroshiro128 + ^52,53^ PRNG that is used in StochasticRounding.jl.

**Figure 3:**
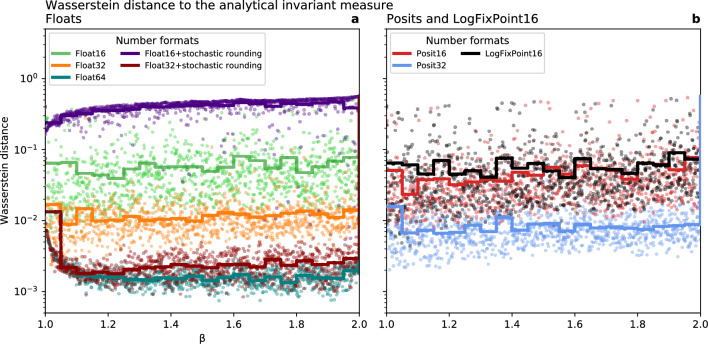
Agreement between the simulated and analytical invariant measures in the generalised Bernoulli map quantified by the Wasserstein distance. For all values $1 \le\beta < 2$ a higher precision number format yields a better agreement with the analytical Bernoulli map. Simulations using (**a**) Floats with and without stochastic rounding, (**b**) Posits and logarithmic fixed-point numbers. The Wasserstein distances are calculated for the invariant measures obtained from $N={10}^{3}$ simulations for each value of $\beta$. Scatter points denote individual Wasserstein distances, solid lines indicate averages across a range of $\beta$ as indicated by steps.

The agreement of the analytical and simulated invariant measures is quantified with the Wasserstein distance (section Methods). For $\beta = 2$ the analytical invariant measure is the uniform distribution $U(0,1)$, whereas all float and posit formats for both deterministic and stochastic rounding simulate a collapse of the attractor to zero such that the invariant measure is the Dirac delta distribution (Fig. 3). The Wasserstein distance $W_{1}$ is in all these cases $W_{1} = 0.5$ and does not improve with precision. However, as previously mentioned, the rounding errors from logfixs prevent a collapse such that for LogFixPoint16 the invariant measure is much better approximated, with $W_{1} = 0.05$.

For $1 \le \beta < 2$ the Wasserstein distance is always reduced going to higher precision, supporting the inference that only the case $\beta = 2$ (and other even integers) presents a pathology where higher precision does not improve the simulated invariant measure arising from the generalised Bernoulli map. The Wasserstein distances of Float32 with stochastic rounding are similarly low to Float64 and no significant difference can be found. However, using stochastic rounding with Float16 is worse than deterministic rounding as here the stochasticity makes it possible that the invariant measure of the generalised Bernoulli map collapses to 0, which is a fixed point (Figure S5). For Float32 the probability of such an occurrence is low and is not observed here. With Float16 most simulations collapse within a few thousand iterations transforming their invariant measures into Dirac distributions. Whether this problem generalises to other systems is questionable. We suspect that this may be a feature of low-dimensional dynamics and may not arise commonly in higher dimensional systems: There the chance of a stochastic perturbation onto a fixed point becomes vanishingly small even at very low precision. Other natural systems do not have fixed points due to time-dependent forcing. The posit format is slightly better than floats at both 16 and 32-bit, as expected from the slightly higher precision.

## Orbits in the Lorenz 1996 system

In contrast to the 1-variable generalised Bernoulli map, most continuous natural systems are simulated numerically with as many variables as computationally affordable by increasing resolution and/or complexity. Weather forecast and climate models often use millions of independent variables that result from a discretisation of continuous variables on the globe. While short periodic orbits in low precision are problematic in the simulation of few-variable systems as discussed in the previous sections, this section tests the hypothesis that large systems are unaffected for all practical purposes.

**Figure 4:**
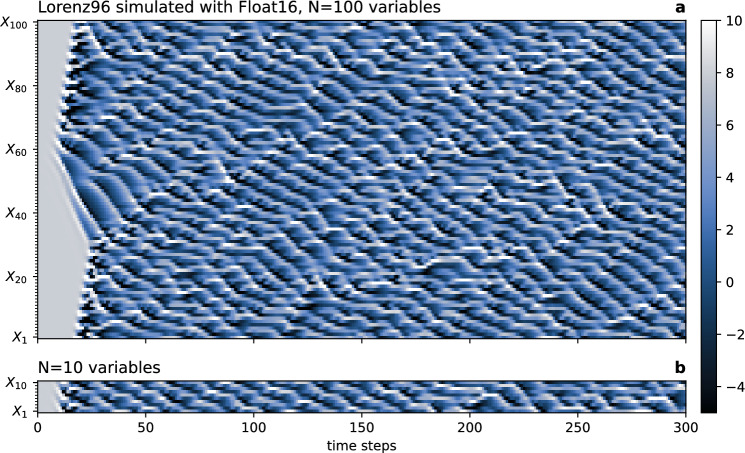
The Lorenz 1996 system simulated with Float16 arithmetic. A Hovmoeller diagram visualising the temporal evolution of every variable ${X}_{i}$ encoded in colour. (**a**) $N = 100$ variables starting from equilibrium ${X}_{i}=8$ (coloured as grey) with a small perturbation in ${X}_{50}$, and (**b**) same as (**a**) but $N=10$ variables, starting with a perturbation in ${X}_{5}$. Pixels visible in the shading represent individual variables and time steps.

To investigate the dependence of periodic orbits on the number of variables in the system we consider the chaotic Lorenz 1996 system^54,55^. This system has been widely studied for data assimilation^56^ and machine learning^57,58^. With *N* variables $X_{i} ,i = 1, \ldots ,N$ the one-layer version is a system of coupled ordinary differential equations.

7 $$\frac{{dX_{i} }}{dt} = X_{i - 1} \left( {X_{i + 1} - X_{i - 2} } \right) - X_{i} + F$$

in a one-dimensional spatial domain with periodic boundary conditions, $X_{N + 1} = X_{1}$ etc. The term $X_{i - 1} \left( {X_{i + 1} - X_{i - 2} } \right)$ implements nonlinear advection, and drag is represented with the relaxation term –*X*_*i*_. The forcing *F* is the single parameter in the Lorenz 1996 system fixed at the common default *F* = 8 which produces chaotic solutions. The forcing is steady in time and constant in space. The system exhibits dynamics of nonlinear wave-wave interactions (Fig. 4a), which are reasonably independent of the number of variables (Fig. 4b). The system can be integrated with as little as $N = 4$ variables without an obvious degradation of the simulated dynamics.

The initial conditions are in equilibrium $X_{i} = F,i \ne j~\forall i$ with only a single variable which is slightly perturbed $X_{j} = F + 0.005 + 0.01\varepsilon$ with $\varepsilon \sim U(0,1)$, drawn from a random uniform distribution in $\left[ {0,1} \right)$. Due to the periodic boundary conditions and the spatially constant forcing the system is spatially invariant. After some several hundred time steps, the information from the initial conditions is removed through the chaotic dynamical evolution (Fig. 4a). The invariant measure µ of one variable $X_{i}$ is therefore identical to that of any other ${\upmu }\left( {X_{i} } \right) = {\upmu }\left( {X_{j} } \right){ }\forall i,j$, as will be further discussed below.

The Lorenz 1996 system is discretized in time using the 4-th order Runge–Kutta scheme^59^ with a time step of ${\Delta }t = 0.1$. At this temporal resolution the system can also be integrated using a low-precision number format such as Float16 (Fig. 4). For more details and a software implementation see Lorenz96.jl^60^. Time integration scheme and time step also have a large impact on chaotic trajectories and hence on the periodic orbit analysis presented here. Numerical stability and performance usually dictate this choice, but low precision can add an additional constraint: Using a shorter time step can cause stagnation as tendencies are too small to be added in the time integration. Stagnation from low precision can be overcome with a compensated time integration^61^ or with stochastic rounding^32^.

### Longer orbits with more variables

**Figure 5:**
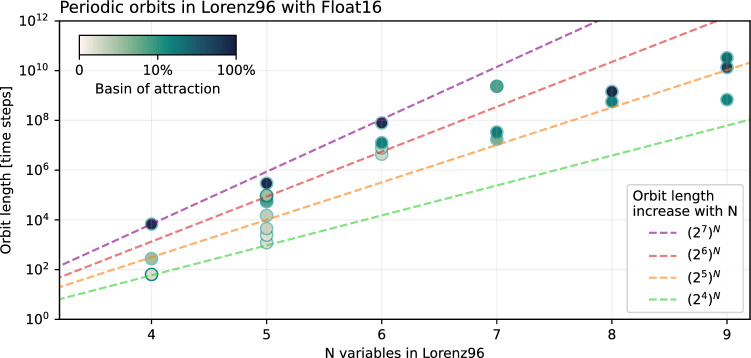
Periodic orbits in Lorenz96 simulated with Float16 with an increasing number of variables. Initial conditions are randomly taken from a high-precision simulation. Basins of attraction (shading) correspond to the share of initial conditions that converge to the respective periodic orbit. Dashed lines provide an orientation for the exponential increase in orbit lengths with the number of variables.

Using $N=4$ variables in the Lorenz 1996 system simulated with Float16, the longest periodic orbit we find is 6756 time steps long (Fig. 5 and Table S1). The basin of attraction is about 0.82, meaning that about 82% of the randomly chosen initial conditions converge onto this orbit. Increasing the number of variables to $N=5$, the longest periodic orbit found increased to a length of 294,995 time steps at a similarly large basin of attraction. For $N > 9$ the orbit search becomes computationally very demanding and requires more than several days on sizable compute clusters with 100 cores (see section Methods for a description of how the orbit search is parallelised). For $N=9$ though, the longest periodic orbit we were able to find has a period of 32,930,252,532 time steps. For a list of periodic orbits found in the Lorenz 1996 system and their respective minimums see Table S1.

### More variables instead of higher precision

In most cases between 4 and 9 variables in the Lorenz 1996 system, the longest orbit is also the one with the largest basin of attraction. The longer the orbit the larger the occupied state space of possible values the variables ${X}_{i}$ can take at a given precision. Consequently, the assumption is that it is most likely that a given trajectory ends up on the longest orbit. However, we also found a counter example as the longest orbit with $N=8$ variables is shorter than the longest with 9 variables (Fig. 5).

The orbit length increases approximately exponentially following a scaling of about ${16}^{N}$ to ${128}^{N}$ from $N=4$ to $N=9$. Such an exponential increase translates to about 4 to 7 effective bits of freedom (as ${2}^{4}=16,{2}^{7}=128$) for every additional variable in Lorenz 1996 represented with Float16. However, the computational resources limit the orbit search for larger $N$, making it hard to constrain this exponential scaling further. Assuming a similar exponential orbit increase holds for larger $N$, extrapolation of these findings suggests orbit lengths on the order of about ${10}^{\mathrm{100,000}}$ for million-variable systems simulated with Float16. This is far beyond the reach of any computational resources currently available. In that sense, while a simulation of such large systems would eventually be periodic, a periodic solution will never be reached.

Longer orbits are promising to avoid periodic solutions in low precision, but short periodic orbits do not necessarily misrepresent a reference invariant measure. We assess the agreement of invariant measures using the Wasserstein distance as before. As a reference invariant measure $\upmu \left({X}_{ref}\right)$ we integrate the Lorenz 1996 system for 1,000,000 time steps with $N=500$ variables using Float64 arithmetic. The Wasserstein distance is then $W\left(\upmu \left({X}_{ref}\right),\upmu \left(X\right)\right)$ with $X$ representing the variables from a Lorenz 1996 simulation with $N$ variables using either Float16 or Float64 arithmetic.

**Figure 6:**
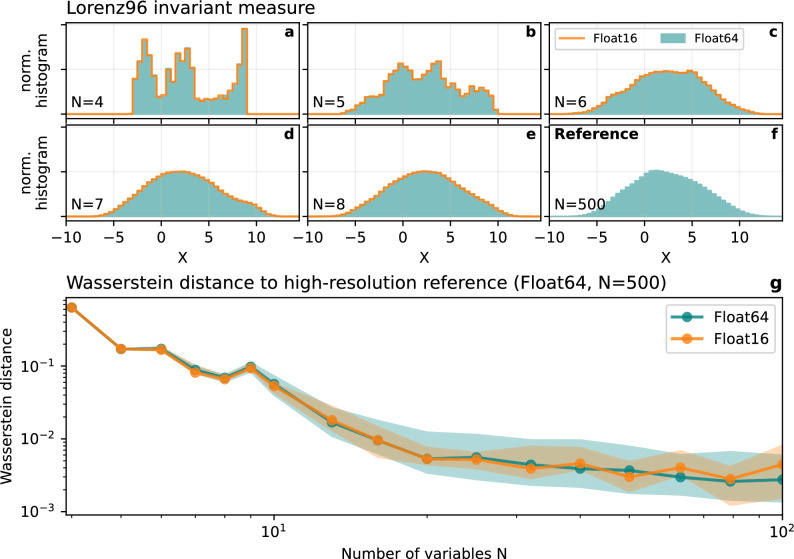
Improvement of the simulated Lorenz 1996 invariant measure with increasing number of variables. (**a**) The invariant measure of Lorenz 1996 simulated with $N=4$ variables. Using Float16 (orange line outlining the histogram) or Float64 (teal filled histogram) arithmetic yields a virtually identical invariant measure. (**b**–**e**) as **(a)** but with an increasing number of variables. (**f**) The reference invariant measure obtained from $N=500$ variables using Float64 arithmetic. (**g**) The Wasserstein distance of the simulated Lorenz 1996 system with respect to the reference. Invariant measures are taken from all available variables, which are invariant due to periodic boundary conditions and spatially-independent forcing (see Eq. 7). Shadings in (**g**) represent the 5–95% confidence interval and solid lines the median obtained from an ensemble simulation with 100 members starting from slightly different random initial conditions.

Using only $N = 4$ variables in the simulation of Lorenz 1996 yields an invariant measure with little resemblance to the reference (Fig. 6a,f), regardless of the number format. While more variables yield an invariant measure that converges to the reference, there is virtually no difference whether Float16 or Float64 arithmetic is used (Fig. 6b–e). The Wasserstein distance significantly reduces with an increasing number of variables, but not with higher precision (Fig. 6g). Given a certain availability of computational resources a better invariant measure is therefore obtained by reducing the precision and reinvesting the performance gain into more variables.

### Discussion

From a rigorous mathematical perspective, we would like to know how the periods of orbits increase with the number of variables and precision. Experimentally, we find orbits that exponentially increase in length with the number of independent variables in the Lorenz 1996 system. Every variable is found to add between 4 and 7 maximum entropy bits that extend orbits by a factor of 2^4^ to 2^7^. Similarly, assuming maximum entropy for additional mantissa bits, the expected length of periodic orbits doubles with every additional mantissa bit in precision.

The Lorenz 1996 system is deemed to be *too easy*^62^ as a toy model for machine learning, meaning that it is too homogeneously chaotic to be actually a challenging problem. In our case, the conclusions from Lorenz 1996 may not translate directly to more chaotic systems: the exponential growth of the periodic orbit length with the number of variables in such systems might be weaker.

For more complex simulations of natural systems such as million-variable climate models, the periodic orbits found here would naively extrapolate to lengths beyond 10^100,000^ time steps even in 16-bit precision. The largest orbits will very likely remain out of reach on future generations of supercomputers, especially if the performance gained from low-precision simulations is reinvested into higher resolution or complexity. In the context of climate models, this supports a vision of low-precision but high-resolution simulations with added stochasticity to accelerate and improve climate predictions.

## Conclusions

We analysed the bifurcation in the logistic map with different number formats and rounding modes. The rounding errors from 16-bit floats, posits or logfixs with deterministic rounding can spuriously stabilise the unstable fixed points. Furthermore, the dense attractors collapse into short periodic orbits. Both issues can be addressed with stochastic rounding. It prevents periodic orbits as a chaotic trajectory is regularly pushed off any periodic orbit due to the stochastic perturbation in rounding. This considerably improves the logistic map bifurcation with Float16.

The periodic orbit spectrum in the generalised Bernoulli map was analysed with different number formats and levels of arithmetic precision. While there are very special cases (such as $\beta = 2$) in which the system’s simulation is greatly degenerated at any precision, in all other cases simulations were found to improve with higher precision. 16 and 32-bit arithmetic result in short periodic orbits in the Bernoulli map, but a sufficiently high precision reduces the error in the invariant measure to a minimum.

Stochastic rounding is also found to be especially beneficial for 32-bit floats in the Bernoulli map. Simulated invariant measures are improved as chaotic trajectories travel with stochastic rounding through otherwise unreachable state space. However, in the Bernoulli map stochastic rounding also causes a non-zero chance that the system collapses on the fixed point. But for any precision higher than Float16 and in systems with more variables this chance quickly vanishes. In many complex natural systems the fixed points are not close to the attractor, further limiting the relevance of this issue in practice.

Increasing the number of variables in the Lorenz 1996 system, we find that more variables improve the simulated invariant measure much more than increased precision with fewer variables does: Doubling the amount of variables yields a more accurate invariant measure than doubling the precision of a high-resolution and high-precision reference. This provides evidence that computational resources should be invested in higher resolution rather than higher precision for the simulation of continuous chaotic systems.

## Methods

### An improved random number generator for uniformly distributed floats

Conventional random number generation for a float $f$ from a uniform distribution $U(0,1)$ uses the following technique: First, 10/23/52 random bits (for Float16/Float32/Float64 respectively) from an unsigned integer are used to set the mantissa bits of floating-point 1. This creates a floating-point number that is uniformly distributed in $\left[\mathrm{1,2}\right)$ as all float formats are uniformly distributed in that range (the exponent bits are constant). Second, 1 is subtracted to obtain a float in $\left[\mathrm{0,1}\right)$, i.e. $f\sim U(1,2)-1$. While this approach is fast, it is statistically imperfect as the resulting distribution $U(1,2)-1$ does not contain all floats in $\left[\mathrm{0,1}\right)$. There are ${2}^{23}$ Float32s in $\left[\mathrm{1,2}\right)$ but the subtraction maps those only to a subset of all $\mathrm{1,065,353,216}\approx {2}^{30}$ Float32s in $\left[\mathrm{0,1}\right)$. This technique only samples from every second float in $\left[\frac{1}{2},1\right)$, every fourth in $\left[\frac{1}{4},\frac{1}{2}\right)$ and so every $2n$-th float in $\left[{2}^{-n},{2}^{-n+1}\right)$. Furthermore, the smallest positive number that can be obtained is about ${10}^{-7}$ for Float32 and ${10}^{-16}$ for Float64. This is many orders of magnitude larger than minpos, the smallest representable positive float, which is about ${10}^{-45}$, ${10}^{-324}$ for Float32, Float64, respectively.

We therefore developed a statistically improved conversion from a random unsigned integer to a uniformly distributed float in $\left[\mathrm{0,1}\right)$. Counting the number of leading zeros $l$ of a random unsigned integer yields $l=0$ at probability $\frac{1}{2}$, $l=1$ at probability $\frac{1}{4}$ and $l=k$ at probability ${2}^{-k-1}$. These probabilities correspond exactly to the share of power-2 exponents in the unit range $\left[\mathrm{0,1}\right)$. Consequently, we translate the number of leading zeros $l$ to the respective exponent bits and use the remaining bits of the unsigned integers for the mantissa bits. The statistical flaws from the conventional conversion as presented above are avoided, but for practical reasons the smallest float that can be sampled is about ${10}^{-20}$ for both Float32 and Float64. It is therefore practically impossible to sample a zero with this technique, which is in contrast to the conventional technique. Just as the chance of obtaining a zero in $[\mathrm{0,1})$ is 0 for real numbers, the method here also has a zero chance for floats. We implement this method in the software package RandomNumbers.jl and use it throughout this study.

### Monte Carlo orbit search

For the generalised Bernoulli map, the random number generator described above is used to sample from all floats in $[\mathrm{0,1})$ to obtain a representative subset of all initial conditions. While there is no guarantee that all orbits are found, those found have the largest basin of attraction. While it is easily possible to miss a periodic orbit, those missed are expected to have a very small basin of attraction and therefore a negligible contribution to the invariant measure. Estimating the invariant measure from these orbits is therefore also expected to be an unbiased approximation that converges to the exact invariant measure. The exact invariant measure is obtained by finding all simulated orbits and their exact basins of attraction rather than using a random set of initial conditions. We verify this methodology for Float16 and Float32, where the exact invariant measure can be calculated in Figure S1. While we cannot find all orbits with Float64, the Monte Carlo-based invariant measure converges to the analytical invariant measure and is for the same sample size a better approximation than using Float16 or Float32. Despite the high precision, a Float64 simulation of the generalised Bernoulli map still substantially degrades some properties of the analytical system: The topological entropy, measuring how trajectories diverge onto distinct orbits, is positive in the analytical system, representing chaotic solutions. However, even with the high precision of Float64 the topological entropy is negative, as trajectories eventually converge onto periodic orbits.

For the Lorenz 1996 system, the space of all possible initial conditions is much larger than the space the attractor occupies. It is therefore more efficient to only choose initial conditions randomly that are already part of, or at least close to, the attractor. The basin of attraction here means therefore the relative share of the initial conditions from the attractor that end up on a given orbit, and not from all possible initial conditions. To obtain an initial condition for the Lorenz 1996 system, one first starts a high-precision simulation from a given initial condition including a small stochastic perturbation (see section "Orbits in the Lorenz 1996 system" for more details). After disregarding a spin-up the information about the chosen initial condition is removed and the stochastic perturbation grows into a fully independent random initial condition. Converting a random time step from a high-precision simulation into the given number format is then intended to emulate sampling from the invariant measure at low precision.

### Efficient orbit search with distributed computing

To find an orbit in a simulation, Eq. (1) is used after every time step to check for equality with a previous time step. However, before an orbit is found, it is unknown whether a given initial condition ${X}_{{t}_{0}}$ is already part of the orbit or still part of the trajectory that is yet to converge onto an orbit. It is possible to use the last time step of a very long spin-up simulation as ${X}_{{t}_{0}}$. This strategy limits the chance that ${X}_{{t}_{0}}$ is not yet part of the orbit, but does not provide a guarantee, nor is it efficient. Instead we implemented a strategy whereby ${X}_{{t}_{0}}$ is updated during simulation and slowly moves forward in time: Updates like ${t}_{0}=round\left(\sqrt{{t}_{1}}\right)$ or ${t}_{0}=round\left(log\left({t}_{1}\right)\right)$, with ${t}_{0}<{t}_{1}$ integers, indicating the time steps, are used. In particular, we use several past time steps of the simulation as ${X}_{{t}_{0}}$ to check for periodicity. Checking for periodicity with *all* past time steps is inefficient as they would have to be stored and $O\left({t}_{1}^{2}\right)$ checks have to be performed in total for all time steps from ${t}_{0}$ to ${t}_{1}$. In contrast, for a constant number of checks per time step, the total number of checks increases only linearly with the simulation time.

Finding $n$ orbits from $n$ different initial conditions is a problem that is parallelizable into $n$ independent processes calculated on $n_p \le n$ processors. We follow ideas of the MapReduce framework: Each worker process starts with a different initial condition and simulates the dynamical system independently of other processes until an orbit is found. This orbit is passed to the main process, which reduces successively all $n$ orbits found into a list of unique orbits, as several initial conditions can yield the same orbit. Instead of defining an orbit by all of the points on it, which would be computationally inefficient for very long orbits, we describe an orbit by the period length and its minimum. The minimum of an orbit is the point for which the ${L}^{2}$ norm is minimised. While it is theoretically possible that an orbit has several minima with identical norms, this occurred rarely in our applications. Two such orbits that are falsely identified as respectively unique are then merged in post-processing. A uniqueness check between two orbits (or one orbit and a list of orbits, in which case the uniqueness check is pairwise against every orbit in the list) is unsuccessful and yields a single orbit only if all of the three following criteria are fulfilled: 1) Length: the two orbits must have the same period length; 2) Minimum norm: the norms of the orbits’ minima have to be identical; 3) Minimum: the orbits’ minima, including possible rotation of the variables for spatially periodic solutions, have to be bitwise identical. While criterion 3 is sufficient to identify the uniqueness of two orbits, it is computationally more efficient to check for criterion 3 only if criterion 2 is fulfilled, which is only checked if criterion 1 is fulfilled, hence the proposed order.

### Wasserstein distance

The invariant measure of a chaotic dynamical system is estimated with histogram binning. To assess the agreement of two histograms representing invariant measures (either simulated or analytical) we use the Wasserstein distance, a metric that derives from the theory of optimal transport, with an ${L}^{1}$ cost. The Wasserstein distance is defined as the least cost at which one can transport all probability mass from histogram $\upmu$ to another histogram $\upnu$, where the cost to move mass $m$ from a bin of $\upmu$ at location $x$ to a bin at location $y$ of $\upnu$ is $m\left|x-y\right|$^33,63^. This gives a non-parametric method to compare probability distributions which accounts for both differences in the probabilities of events as well as their separations in the underlying space, so that closeness in Wasserstein distance truly corresponds to a natural notion of closeness between probability distributions^63^, Thm 7.12.

## Supplementary Information

Supplementary Information.

## Data Availability

The repository BernoulliMap is available at https://github.com/milankl/BernoulliMap^64^ and contains the software to produce the analysis presented here. Lorenz96.jl (v0.3) is available at https://github.com/milankl/Lorenz96.jl^60^. LogFixPoint16s.jl (v0.3) is available at https://github.com/milankl/LogFixPoint16s.jl^41^. StochasticRounding.jl (v0.6) is available at https://github.com/milankl/StochasticRounding.jl^45^.
