# Supplementary material for: Periodic orbits in chaotic systems simulated at low precision
Source: Sci Rep. 2023 Jul 14;13:11410. doi: 10.1038/s41598-023-37004-4 (PMC10349059; doi:10.1038/s41598-023-37004-4)
Supplement: Supplementary file 1 — Supplementary Information. [file 41598_2023_37004_MOESM1_ESM.pdf]

Supplement

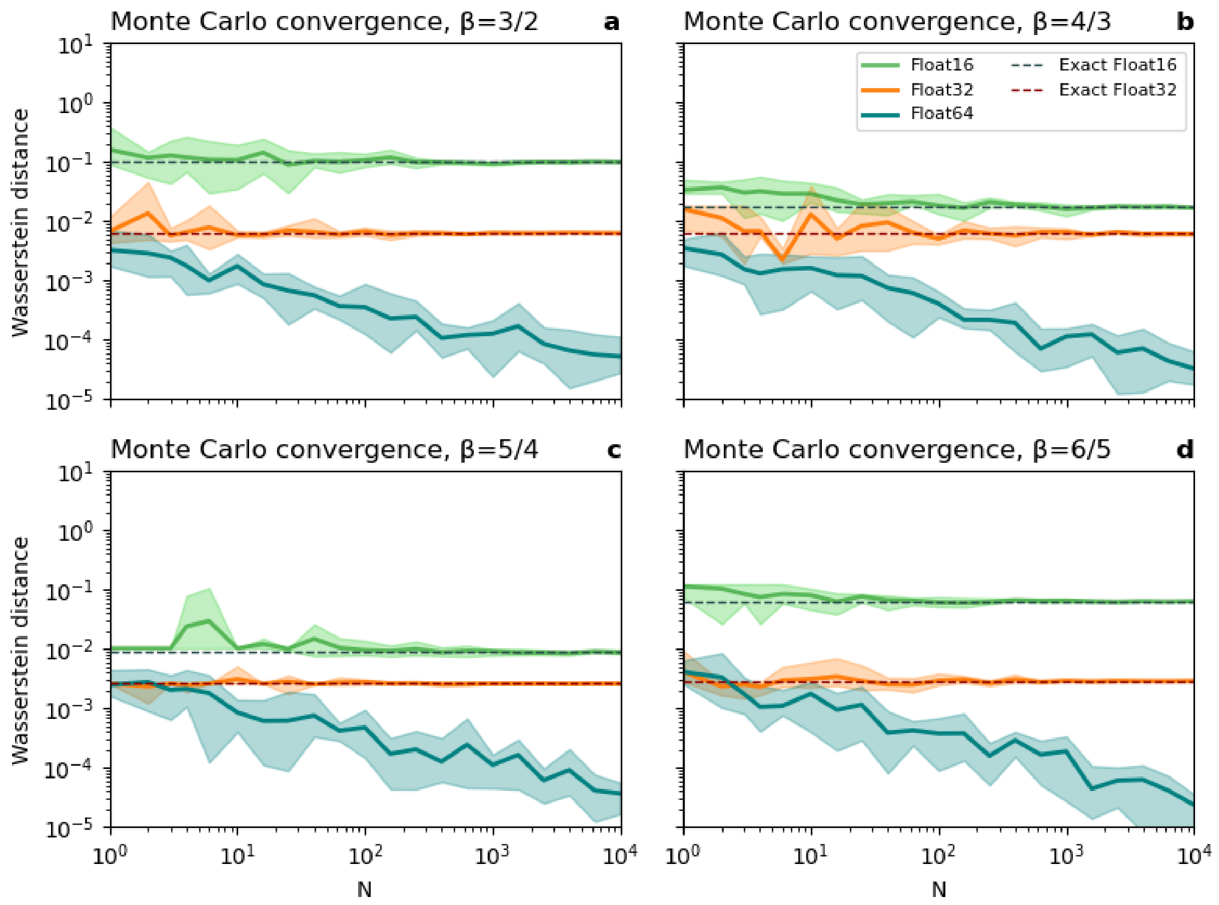

Figure S1 | Convergence of the Monte Carlo sampling to estimate invariant measures. The analytical and simulated invariant measures (Figure S2) from N random initial conditions uniformly distributed in $[0,1)$ are assessed with the Wasserstein distance. About $N=10^3$ random initial conditions allow for a robust estimate of the invariant measure with Float16 and Float32, virtually identical with exact invariant measure obtained from computing all 15,360 Float16 and 1,065,353,216 Float32 numbers in $[0,1)$, respectively. Solid lines represent the mean and shading the min-max range.

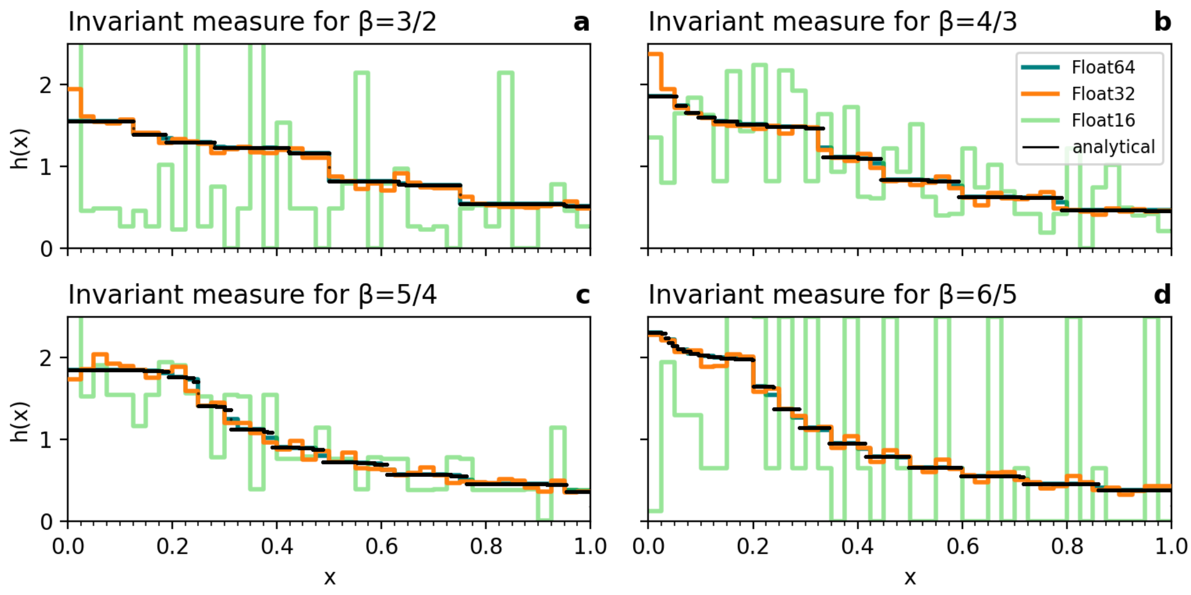

Figure S2 | The invariant measures of the generalised Bernoulli map. The Bernoulli map is simulated with parameter **a** $\beta = \frac{3}{2}$, **b** $\beta = \frac{4}{3}$, **c** $\beta = \frac{5}{4}$, **d** $\beta = \frac{6}{5}$ and calculated with different number formats Float64, Float32 and Float16. The invariant measures of Float16 and Float32 are obtained from periodic orbits found by starting from N = 10,000 initial conditions $x \in [0, 1)$ chosen from a random uniform distribution. For Float64 long integrations (10,000 iterations, disregarding a spin-up of 5,000 iterations) of the Bernoulli map are used instead. Histograms use the bin width 0.025. The analytical invariant measure is not binned, which accounts for the discrepancy to Float64.

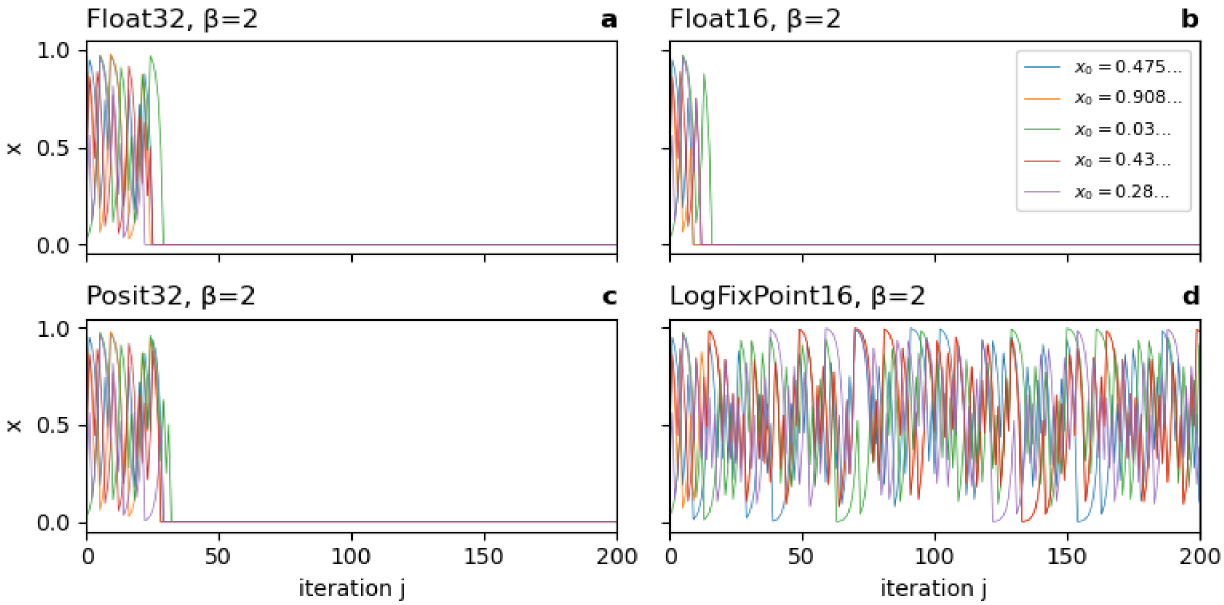

Figure S3 | The Bernoulli map simulated with different number formats. a Float32, **b** Float16, **c** Posit32 and **d** LogFixPoint16. The Bernoulli map does not introduce arithmetic rounding errors in **a-c**, such that stochastic rounding has no impact. Only the initial conditions are subject to rounding. However, with logfix arithmetic the subtraction in the Bernoulli map introduces rounding errors in **d** that prevent the stalling at 0 in **a-c**.

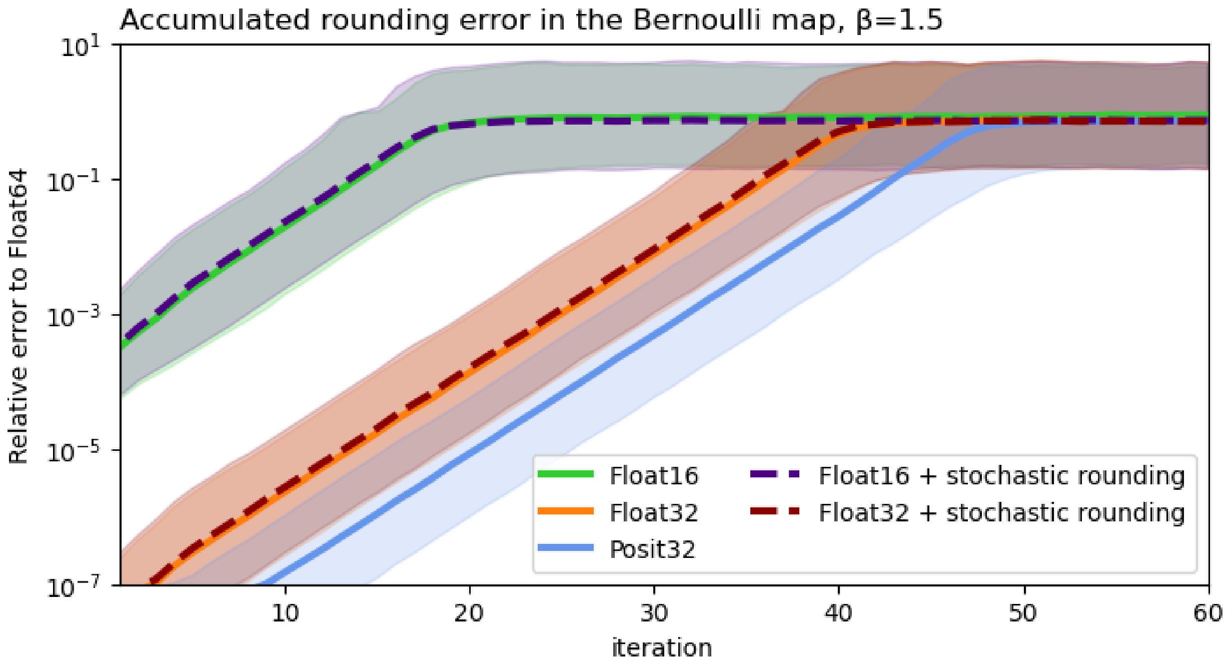

Figure S4 | Accumulated rounding error in the Bernoulli map. Starting from identical $N = 10,000$ random initial conditions, the accumulated rounding error is the relative error of the given number format relative to a Float64 integration. Solid lines represent median errors across all initial conditions, shading the interdecile range. Other choices for β yield a similar comparison between the number formats, but decreasing β towards 1 also decreases the error growth.

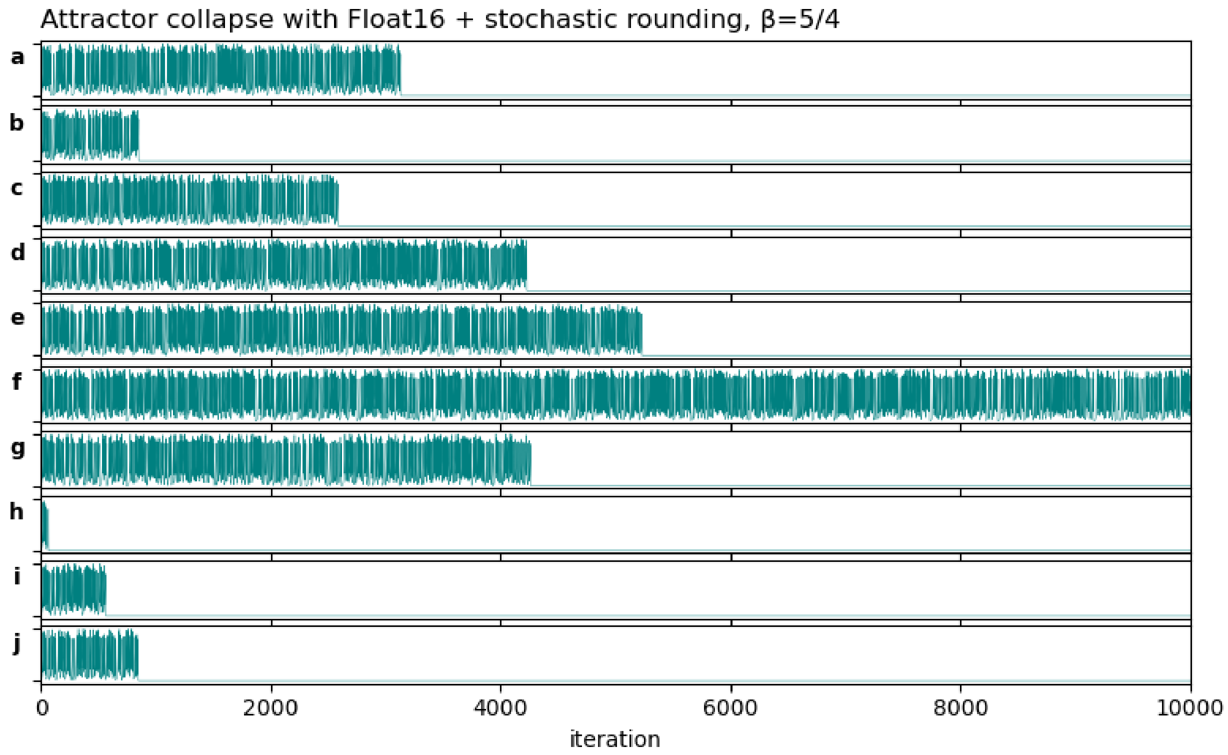

Figure S5 | Attractor collapse of the Bernoulli map with Float16 and stochastic rounding. a-j Simulation of the Bernoulli map starting from identical initial conditions and with $\beta = \frac{5}{4}$, only the state of the random number generator for stochastic rounding differs. After 10,000 iterations only sublot **j** did not yet collapse to 0. The y-axes denote the value of x_i in [0,1].

Period length	basin	minimum
**Float16, N=4**		
64	0.148301	Float16[8.4, -1.172, 1.208, 1.527]
64	0.025185	Float16[1.217, 1.563, 8.38, -1.199]
277	0.011029	Float16[1.031, 1.361, 8.43, -1.286]
6756	0.815485	Float16[8.47, -1.344, 0.783, 1.106]
**Float16, N=5**		
1205	0.0005	Float16[-4.465, 2.676, 1.983, 3.566, 4.45]
2415	0.0003	Float16[0.794, 7.3, -0.1984, -1.412, 0.09503]
4485	0.0019	Float16[2.105, 3.656, 4.258, -4.375, 2.752]
14925	0.0032	Float16[-0.2502, 0.8438, 7.652, -0.1371, -1.393]
53995	0.0047	Float16[-0.14, -1.364, -0.1969, 0.7847, 7.605]
59945	0.0612	Float16[7.586, -0.1426, -1.432, -0.198, 0.8076]
88110	0.0275	Float16[1.212, 7.62, -0.5586, -0.641, 0.1917]
91980	0.0792	Float16[1.065, 7.64, -0.4578, -0.8286, 0.09595]
97215	0.0112	Float16[1.34, 7.562, -0.6274, -0.4536, 0.3428]
294995	0.8103	Float16[-1.157, -0.03723, 0.952, 7.598, -0.3352]
**Float16, N=6**		
4405392	0.001	Float16[-3.361, 0.6084, 1.09, 3.375, 4.805, -0.4067]
7820184	0.002	Float16[1.955, 3.916, 3.021, -4.24, 0.5537, 0.701]
12688470	0.181	Float16[1.222, 1.088, 1.614, 3.6, 3.574, -4.023]
78874782	0.816	Float16[3.219, 4.9, -0.2283, -3.436, 0.3677, 0.713]
**Float16, N=7**		
17531430	0.03	Float16[-0.4985, 2.64, 5.97, -2.188, 0.1743, 0.2306, 0.103]
33926067	0.18	Float16[-0.4238, -0.704, 2.56, 5.92, -1.828, -0.795, 0.8286]
2355085796	0.79	Float16[6.113, -1.858, -0.6133, 0.7876, -0.1544, -0.707, 1.994]
**Float16, N=8**		
569018386	0.1875	Float16[-1.604, 1.616, 2.139, 2.68, -2.373, -0.6445, 0.5273, 3.215]
1449659326	0.8125	Float16[1.264, 1.165, 3.008, -2.305, -1.467, 0.797, 2.666, -2.35]
**Float16, N=9**		
681602535	0.15625	Float16[-3.072, -2.104, -0.4575, -0.08777, 1.392, 0.8486, 1.859, 4.867, 5.086]
13428881973	0.59375	Float16[1.604, 4.234, 4.36, -4.59, -0.2253, 0.362, 1.207, 1.533, 0.793]
32930252532	0.25	Float16[0.769, 1.876, -1.593, -0.4277, -0.3481, 3.348, 6.816, -1.179, -1.096]

Table S1 | Periodic orbits in the Lorenz 1996 system simulated with Float16 and N variables. The period length is given as the number of time steps. The basin is the fraction of initial conditions ending up on a given orbit. The minimum is one point on the orbit for which the L^2 norm is minimised. Simulations performed with Lorenz96.jl.
